# Estimation of the Relative Contribution of Postprandial Glucose Exposure to Average Total Glucose Exposure in Subjects with Type 2 Diabetes

**DOI:** 10.1155/2016/3452898

**Published:** 2016-08-18

**Authors:** Bo Ahrén, James E. Foley

**Affiliations:** ^1^Lund University, 22184 Lund, Sweden; ^2^Novartis Pharmaceuticals Corporation, East Hanover, NJ 07936-1080, USA

## Abstract

We hypothesized that the relative contribution of fasting plasma glucose (FPG) versus postprandial plasma glucose (PPG) to glycated haemoglobin (HbA1c) could be calculated using an algorithm developed by the A1c-Derived Average Glucose (ADAG) study group to make HbA1c values more clinically relevant to patients. The algorithm estimates average glucose (eAG) exposure, which can be used to calculate apparent PPG (aPPG) by subtracting FPG. The hypothesis was tested in a large dataset (comprising 17 studies) from the vildagliptin clinical trial programme. We found that 24 weeks of treatment with vildagliptin monotherapy (*n* = 2523) reduced the relative contribution of aPPG to eAG from 8.12% to 2.95% (by 64%, *p* < 0.001). In contrast, when vildagliptin was added to metformin (*n* = 2752), the relative contribution of aPPG to eAG insignificantly increased from 1.59% to 2.56%. In conclusion, glucose peaks, which are often prominent in patients with type 2 diabetes, provide a small contribution to the total glucose exposure assessed by HbA1c, and the ADAG algorithm is not robust enough to assess this small relative contribution in patients receiving combination therapy.

## 1. Introduction

Glycated haemoglobin (HbA1c) levels reflect the average blood glucose levels over the past 12 weeks including both fasting plasma glucose (FPG) and postprandial plasma glucose (PPG) levels [1]. This “Gold Standard” to assess average glycaemic exposure in the diagnosis and treatment of type 2 diabetes, in individual patients as well as in population studies, is well established. “The independent contribution of PPG excursions to the overall glucose exposure and its role in the development of both micro- and macrovascular complications of diabetes remain subject to continuing debate in type 2 diabetes” [2].

There has not been universal agreement regarding nature of these excursions. In addition to exposure per se there is also the concept that high peak glucose levels per se are pathological, leading to increased cardiovascular (CV) risk. However, the associations between CV risk and PPG are based on 2-hour oral glucose tolerance test (OGTT) values [3]. It is not possible to determine whether these values are due to the peak values at 2 hours or a reflection of the PPG exposure on top of the underlying FPG exposure (peak value versus area under the curve). They have generally been assumed to be a reflection of the peak glucose values and this concept has expanded to include the notion of glucose variability which includes not only the hyperglycaemic amplitude but also the hypoglycaemic amplitude. More recently 24-hour continuous glucose monitoring (CGM) has been utilized to assess glucose variability. In order to summarize variability from the vast amount of data collected by CGM, a variety of parameters such as the mean amplitude of glucose excursions (MAGE) and coefficient of variation (SD) have been calculated [4]. Calculating MAGE or SD from CGM measurements is not feasible for routine clinical practice, but a number of approaches that could be amenable to routine clinical practice have been suggested to assess glucose variability such as a one-hour OGTT [5] and four-point preprandial self-monitoring of blood glucose [6]. The focus of this paper however is restricted to the contribution of the PPG on the total glucose exposure rather than on these assessments of glucose variability.

The data from Monnier et al. indicate that the relative PPG exposure is larger at lower HbA1c levels than at high HbA1c levels, but that the overall contribution to the total exposure is still rather small [2]. In contrast, a recent meta-analysis of 11 studies found that there was a better correlation between PPG and HbA1c than between FPG and HbA1c [7] suggesting that PPG exposure is more closely reflecting the HbA1c than is the FPG exposure. This makes it difficult to reconcile with the data from Monnier et al. indicating that the PPG exposure is a much smaller percent of the total exposure relative to the FPG exposure. Closer examination of the data however leads to a different conclusion. In this meta-analysis of 11 studies the PPG is assessed by 2-hour values after nonstandardized breakfast meal tests. These 2-hour values likely are a reflection of the PPG exposure on top of the underlying FPG exposure. Thus it follows that the sum of FPG and PPG exposure would be a better reflection of the total exposure as assessed by HbA1c than would be the FPG alone. However, the 2-hour values after nonstandardized breakfast meal tests are a rather indirect and not a very quantitative approach to assessing the PPG exposure; thus we asked the question of whether one can get a more quantitative direct assessment of the PPG exposure. It occurred to us that if the HbA1c exposure could be expressed in the same units as the FPG, then the apparent PPG exposure could be calculated from the difference.

HbA1c levels have traditionally been reported in terms of percentage (%) values; however, recently HbA1c values have also been reported in units of mmol/mol in an effort to make the HbA1c values more clinically relevant to patients. This is possible after the A1c-Derived Average Glucose (ADAG) study group developed an algorithm to calculate the estimated average glucose (eAG) exposure (in mmol/mol) based on HbA1c, by combining the weighted results from at least two days of CGM performed four times with seven-point daily self-monitoring of capillary glucose performed at least three days/week in a large number of patients. These values were then integrated and related to % HbA1c using a linear regression analysis (*r* ^2^ = 0.84, *p* < 0.0001) [8]. Based on this calculation, eAG can also be determined in units of mmol/L using standardized linear regression: eAG_mmol/L_ = ([{28.7 × HbA1c%} − 46.7] ÷ 18) [8].

Using this algorithm, it would be possible to calculate the eAG in units of mmoles/L, which can be used to calculate the apparent postprandial glucose (aPPG), further allowing an estimation of its contribution to HbA1c. We have analysed this possibility by calculating the ADAG eAG and aPPG values by subtracting FPG from eAG. The aim of this analysis was to evaluate whether the approach of calculating aPPG from eAG could be used to determine the relative contributions of FPG versus aPPG exposure relative to eAG exposure.

## 2. Methods

For the current analysis, datasets from 17 studies using vildagliptin monotherapy (50 mg once daily [qd] or twice daily [bid]) or vildagliptin plus metformin combination therapy in the vildagliptin clinical trial programme were pooled (Supplementary Table S1, available online at http://dx.doi.org/10.1155/2016/3452898). The eAG (in mmol/L) was calculated from HbA1c values using standardized linear regression (eAG_mmol/L_ = [{28.7 × HbA1c%} − 46.7] ÷ 18) [8], and aPPG values were derived by subtracting FPG from eAG (aPPG = eAG − FPG). The relative contributions of FPG and aPPG to eAG were compared before and after 24 weeks of treatment with vildagliptin 50 mg qd/bid in drug-naïve patients and in those previously treated with metformin.

### 2.1. Statistical Analysis

Patient demographic and baseline characteristics and mean changes in HbA1c, eAG, and FPG were summarized descriptively. Individual linear regression analyses were performed to assess the effects of various factors (age, duration of disease, body mass index [BMI], and eAG) on changes in aPPG after 24 weeks of treatment. The differences between means were evaluated using Student's *t*-test.

## 3. Results

Data from 5275 patients were analysed: 2523 in the vildagliptin monotherapy group and 2752 in the vildagliptin plus metformin group. Baseline patient characteristics are summarized in Table 1. As expected patients in the vildagliptin monotherapy group were several years younger and had diabetes for a shorter duration compared with those in the vildagliptin plus metformin group. Interestingly, the monotherapy group had higher HbA1c levels but similar FPG values compared with those in the vildagliptin plus metformin group.

Concerning vildagliptin monotherapy, after 24 weeks of treatment, vildagliptin monotherapy reduced (mean ± standard error [SE]) HbA1c by −1.11 ± 0.03% (*p* < 0.001) and FPG by −1.12 ± 0.05 mmol/L (*p* < 0.001). The corresponding changes in eAG were  −1.77 ± 0.04 mmol/L (*p* < 0.001); subtracting FPG from the eAG resulted in a 0.64 ± 0.04 mmol/L reduction in the aPPG (*p* < 0.001). At baseline, the relative contribution of aPPG to eAG was 8.12%. After treatment, the relative contribution of aPPG to eAG was 2.95%, corresponding to an observed decrease of almost 64% (*p* < 0.001). The changes in aPPG correlated weakly with age (*r* ^2^ = 0.0055, *p* = 0.0002), duration of diabetes (*r* ^2^ = 0.0021, *p* = 0.0204), and baseline eAG (*r* ^2^ = 0.0111, *p* < 0.0001) but not with BMI (*r* ^2^ = 0.0002, *p* = 0.4843).

Concerning vildagliptin plus metformin combination therapy, after 24 weeks of treatment, vildagliptin plus metformin combination therapy reduced (mean ± SE) HbA1c by −0.88 ± 0.02% (*p* < 0.001) and FPG by  −1.46 ± 0.04 mmol/L (*p* < 0.001). The corresponding changes in eAG were  −1.40 ± 0.03 mmol/L (*p* < 0.001); subtracting FPG from the eAG resulted in only a 0.05 ± 0.04 mmol/L reduction (*p* < 0.001). At baseline, the relative contribution of aPPG to eAG was only 1.59%. Thus when patients were on stable dose of metformin, unexpectedly and in contrast to the monotherapy group, there was essentially no difference between eAG and FPG. Furthermore, there was an insignificant mean increase as opposed to an expect decrease in the relative contribution of aPPG to eAG (2.56%) after 24 weeks of treatment.

## 4. Discussion

In this paper we have utilized A1c-Derived Average Glucose (ADAG) study group algorithm to calculate the estimated average glucose (eAG) exposure in units of mmol/L. We then subtracted the FPG from eAG in order to calculate the apparent postprandial glucose (aPPG) exposure. This allowed us then to estimate the PPG exposure contribution to total glucose exposure.

Initially we carried this analysis out on large pool of monotherapy patients. We found that the contributions of aPPG relative to eAG at baseline (~8%) and after 24 weeks of treatment with vildagliptin monotherapy (~3%) were similar to those that can be estimated from our meal assessments of PPG; we calculated the above baseline glucose areas under curve values performed during standard breakfast meals (SBM) in a representative study [9] and then tripled these areas under the curves to estimate the daily PPG exposure. This suggested to us that, in this study population, the ADAG algorithm for estimation of aPPG and eAG could provide a quantitative estimate of the 24-hour PPG exposure.

We then applied this approach to our large pool of patients who had been stable but inadequately controlled on metformin. In contrast, the contributions of aPPG to eAG at baseline (<2%) and after 24 weeks of treatment with vildagliptin plus metformin combination therapy (~0%) were markedly below the estimated PPG exposure obtained from glucose values recorded during SBM in a representative study [10], suggesting that the glucose exposure algorithm is not robust enough to provide an estimate of the 24-hour PPG exposure when vildagliptin was added to metformin. We believe that in light of the large sample size the difference between the two groups is not by chance. There were differences between the two treatment groups; the monotherapy patients were not always stable on diet and exercise when vildagliptin monotherapy was initiated, whereas the metformin patients were on a stable dose of metformin when vildagliptin was initiated; and although the monotherapy group started from a higher HbA1c level (8.4% versus 7.9%), there was no difference in the FPG between the two groups. We cannot rule out that the FPG is less reliable in the presumably less stable monotherapy group, but with such a large sample size it is difficult to conceive of how this would explain the way the ADAG eAG algorithm predicts PPG exposure in vildagliptin monotherapy group but not in patients receiving combination therapy with metformin. The apparent lower contribution of PPG exposure in the metformin treated patients is unexpected since one would normally expect to see the PPG exposure relative to the total exposure greatest at lower levels of glycaemia [2]. Once again with such a large sample size this cannot be a chance finding. The data suggest that metformin is having a greater effect on PPG exposure relative to FPG than previously appreciated. This could be secondary to improvement of hepatic insulin signalling which may reduce postprandial hepatic glucose delivery [11] or metformin's effect to reduce food consumption [12].

Recently the applicability of the ADAG eAG was evaluated in older adults with diabetes. The authors calculated mean glucose values from the CGM data from 90 patients with mean age of 76 years and with mean HbA1c values of 7.9% and calculated the ADAG eAG from the HbA1c values; the relationship between HbA1c and mean glucose values from the CGM data was different from the relationship between HbA1c and eAG indicating that A1c-Derived Average Glucose (ADAG) study group algorithm to calculate eAG may not accurately reflect average glucose in older adults [13]. This previous report of older adults coupled with the current unexpected findings in patients on metformin treatment suggests that the care should be taken not to overrate the robustness of the eAG.

## 5. Conclusion

The results of this analysis in no way challenge the use of the ADAG glucose exposure algorithm for its intended purpose to provide a more meaningful expression of the total 24-hour glucose exposure [8]. This analysis does support, however, the notion that although glucose peaks are often very prominent in patients with type 2 diabetes, their contribution to the total glucose exposure assessed by HbA1c is rather small. We conclude that the ADAG glucose exposure algorithm is not robust enough across all patient subgroups to assess the small relative contribution of PPG exposure to the total glucose exposure.

## Supplementary Material

The supplementary material contains a table which includes the sample size of monotherapy and of vildagliptin plus metformin patients from each of the clinical studies included in the vildagliptin clinical trial programme pool. The references to each of the published studies follows the table.

## Figures and Tables

**Table 1:** Patient demographics and baseline characteristics.

	Vildagliptin monotherapy (*n* = 2523)	Vildagliptin/metformin combination therapy (*n* = 2752)
Age (years)	54.7 ± 0.2	56.8 ± 0.2
Gender: male, *n* (%)	1435 (48)	1497 (52)
BMI (kg/m^2^)	30.8 ± 0.1	31.5 ± 0.1
Diabetes duration (years)	2.3 ± 0.1	5.2 ± 0.1
HbA1c (%)	8.4 ± 0.02	7.9 ± 0.02
eAG (mmol/L)	10.8 ± 0.03	10.0 ± 0.03
FPG (mmol/L)	9.9 ± 0.1	9.8 ± 0.1
aPPG (mmol/L)	0.9 ± 0.04	0.2 ± 0.04

Data are presented as mean ± SE unless otherwise stated.

aPPG, apparent postprandial glucose; BMI, body mass index; eAG, estimated average glucose; FPG, fasting plasma glucose; HbA1c, glycated haemoglobin; SE, standard error.

## Abbreviations

HbA1: Glycated haemoglobin
FPG: Fasting plasma glucose
PPG: Postprandial plasma glucose
CV: Cardiovascular
OGTT: Oral glucose tolerance test
CGM: Continuous glucose monitoring
MAGE: Mean amplitude of glucose excursions
SD: Coefficient of variation
ADAG: A1c-Derived Average Glucose
eAG: Estimated average glucose
aPPG: Apparent postprandial glucose
SE: Standard error.
