# Supplementary material for: Estimation of the Relative Contribution of Postprandial Glucose Exposure to Average Total Glucose Exposure in Subjects with Type 2 Diabetes
Source: Int J Endocrinol. 2016 Aug 18;2016:3452898. doi: 10.1155/2016/3452898 (PMC5007300; doi:10.1155/2016/3452898)
Supplement: Supplementary file 1 — The supplementary material contains a table which includes the sample size of monotherapy and of vildagliptin plus metformin patients from each of the clinical studies included in the vildagliptin clinical trial programme pool. The references to each of the published studies follows the table. [file 3452898.f1.pdf]

Supporting information

TABLE S1: Sample size in clinical studies from vildagliptin clinical trial programme, monotherapy and vildagliptin plus metformin.

Study	Vildagliptin 50 mg qd/bid (n=2523)	Vildagliptin 50 mg + metformin (n=2752)
1	5	-
2	200	-
3	135	-
4	436	-
5	469	-
6	58	-
7	393	-
8	431	-
9	6	-
10	14	-
11	132	-
12	244	421
13	-	145
14	-	112
15	-	1363
16	-	449
17	-	262

bid, twice daily; qd, once daily

Supplementary references

1. S. Ristic, S. Byiers, J. Foley, and D. Holmes, "Improved glycaemic control with dipeptidyl peptidase-4 inhibition in patients with type 2 diabetes: vildagliptin (LAF237) dose response," *Diabetes Obesity and Metabolism,* vol. 7, no. 6, pp. 692–698, 2005.

2. S. Dejager, S. Razac, J. E. Foley, and A. Schweizer, "Vildagliptin in drug-naïve patients with type 2 diabetes: a 24-week, double-blind, randomized, placebo-controlled, multiple-dose study," *Hormone and Metabolic Research,* vol. 39, no. 3, pp. 218–223, 2007.

3. W. A. Scherbaum, A. Schweizer, A. Mari et al., "Efficacy and tolerability of vildagliptin in drug-naïve patients with type 2 diabetes and mild hyperglycaemia*," *Diabetes Obesity and Metabolism,* vol. 10, no. 8, pp. 675–682, 2008.

4. A. Schweizer, A. Couturier, J. E. Foley, and S. Dejager, "Comparison between vildagliptin and metformin to sustain reductions in HbA(1c) over 1 year in drug-naïve patients with Type 2 diabetes," *Diabetic Medicine,* vol. 24, no. 9, pp. 955–961, 2007.

5. J. E. Foley and S. Sreenan, "Efficacy and safety comparison between the DPP-4 inhibitor vildagliptin and the sulfonylurea gliclazide after two years of monotherapy in drug-naïve patients with type 2 diabetes," *Hormone and Metabolic Research,* vol. 41, no. 12, 905–909, 2009.

6. H. Krum, V. Lukashevich, G. B. Bolli, P. Kozlovski, W. Kothny, and P. Ponikowski, "No significant difference in risk of heart failure hospitalization with vildagliptin in diabetic patients with systolic chronic heart failure: VIVIDD study," *Diabetes,* vol. 63, Supplement 1, pp. A743, 2014.

7. C. Pan, W. Yang, J. P. Barona et al., "Comparison of vildagliptin and acarbose monotherapy in patients with Type 2 diabetes: a 24-week, double-blind, randomized trial," *Diabetic Medicine,* vol. 25, no. 4, pp. 435–441, 2008.

8. J. Rosenstock, M. A. Baron, S. Dejager, D. Mills, and A. Schweizer, "Comparison of vildagliptin and rosiglitazone monotherapy in patients with type 2 diabetes: a 24-week, double-blind, randomized trial," *Diabetes Care,* vol. 30, no. 2, pp. 217–223, 2007.

9. W. D. Strain, V. Lukashevich, W. Kothny, M. J. Hoellinger, and P. M. Paldánius, "Individualised treatment targets for elderly patients with type 2 diabetes using vildagliptin add-on or lone therapy (INTERVAL): a 24 week, randomised, double-blind, placebo-controlled study," *Lancet,* vol.382, no. 9890, pp. 409-416, 2003.

10. J. E. Foley, M. C. Bunck, D. L. Möller-Goede et al., "Beta cell function following 1 year vildagliptin or placebo treatment and after 12 week washout in drug-naïve patients with type 2 diabetes and mild hyperglycaemia: a randomised controlled trial," *Diabetologia,* vol. 54, no. 8, pp. 1985–1991, 2011.

11. F. X. Pi-Sunyer, A. Schweizer, D. Mills, and S. Dejager, "Efficacy and tolerability of vildagliptin monotherapy in drug-naïve patients with type 2 diabetes," *Diabetes Research and Clinical Practice,* vol. 76, no. 1, pp. 132–138, 2007.

12. E. Bosi, F. Dotta, Y. Jia, and M. Goodman, "Vildagliptin plus metformin combination therapy provides superior glycaemic control to individual monotherapy in treatment-naïve patients with type 2 diabetes mellitus," *Diabetes Obesity and Metabolism,* vol. 11, no. 5, pp. 506–515, 2009.

13. E. Bosi, R. P. Camisasca, C. Collober, E. Rochotte, and A. J. Garber, “Effects of vildagliptin on glucose control over 24 weeks in patients with type 2 diabetes inadequately controlled with metformin,” *Diabetes Care,* vol. 30, no. 4, pp. 890–895, 2007.

14. C. Pan, X. Xing, P. Han et al., “Efficacy and tolerability of vildagliptin as add-on therapy to metformin in Chinese patients with type 2 diabetes mellitus,” *Diabetes Obesity and Metabolism,* vol. 14, no. 8, pp. 737–744, 2012.

15. E. Ferrannini, V. Fonseca, B. Zinman et al., “Fifty-two-week efficacy and safety of vildagliptin vs. glimepiride in patients with type 2 diabetes mellitus inadequately controlled on metformin monotherapy,” *Diabetes Obesity and Metabolism,* vol. 11, no. 2, pp. 157–166, 2009.

16. C. Filozof and J. F. Gautier, “A comparison of efficacy and safety of vildagliptin and gliclazide in combination with metformin in patients with type 2 diabetes inadequately controlled with metformin alone: a 52-week, randomized study,” *Diabetic Medicine,* vol. 27, no. 3, pp. 318–326, 2010.

17. G. Bolli, F. Dotta, L. Colin, B. Minic, and M. Goodman, “Comparison of vildagliptin and pioglitazone in patients with type 2 diabetes inadequately controlled with metformin,” *Diabetes Obesity and Metabolism,* vol. 11, no. 6, pp. 589–595, 2009.
